# Supplementary material for: Composition and thermal processing evaluation of yeast ingredients as thiamin sources compared to a standard vitamin premix for canned cat food
Source: PLoS One. 2022 Aug 2;17(8):e0271600. doi: 10.1371/journal.pone.0271600 (PMC9345363; doi:10.1371/journal.pone.0271600)
Supplement: S1 Table — (DOCX) [file pone.0271600.s001.docx]

| **Table S1: Nutritional composition of ingredients^1^ used to produce canned cat food containing different sources of thiamin.** | | | | | | | | | | | | | | | |
| --- | --- | --- | --- | --- | --- | --- | --- | --- | --- | --- | --- | --- | --- | --- | --- |
| Nutrient | MDC | PL | GC | GG | PC | MP | KC | TA | ST | VE | BR | VP | LBV | BY | EA |
| Moisture, % | 67.3 | 75.2 | 71.7 | 7.9 | 1.0 | 5.8 | 8.9 | 0.4 | 0.7 | 1.3 | 12.5 | 7.2 | 5.4 | 7.7 | 4.7 |
|  | ------------------------------------------------------- Dry matter basis ------------------------------------------------------- | | | | | | | | | | | | | | |
| Thiamin, mg/kg | 1.2 | 9.2 | 1.8 | 3.1 | 5.5 | 1.6 | 1.4 | 2.1 | 2.3 | 15.2 | 1.6 | 17933.3 | 1271.7 | 30.1 | 24.9 |
| Crude protein, % | 41.4 | 67.7 | 46.2 | 4.8 | 0.7 | 11.5 | 3.0 | 71.7 | 0.6 | 1.4 | 9.2 | 8.7 | 56.4 | 51.6 | 52.4 |
| Crude fat, % | 50.2 | 24.5 | 43.7 | 0.2 | 0.0 | 1.3 | 0.7 | 0.0 | 0.0 | 53.2 | 0.8 | 7.9 | 0.8 | 0.5 | 2.3 |
| Crude fiber, % | - | - | - | 2.53 | 0.03 | 0.37 | 4.18 | 0.33 | 0.00 | 0.57 | 0.42 | 29.97 | 0.08 | 0.70 | 3.73 |
| Ash, % | 7.04 | 4.78 | 11.06 | 0.86 | 100.67 | 56.97 | 42.70 | 0.65 | 100.50 | 43.87 | 0.70 | 29.17 | 6.19 | 5.65 | 6.37 |
| NFE^2^, % | - | - | - | 91.6 | 0.0 | 29.9 | 49.4 | 27.3 | 0.00 | 1.0 | 88.9 | 24.3 | 36.5 | 41.6 | 35.2 |
| Calcium, % | 2.69 | 0.03 | 4.05 | 0.07 | 0.08 | 0.83 | 0.95 | 0.01 | 0.06 | 0.26 | 0.02 | 7.90 | 0.15 | 0.24 | 0.56 |
| Phosphorus, % | 1.663 | 1.213 | 2.332 | 0.05 | 0.000 | 0.157 | 0.030 | 0.000 | 0.000 | 0.000 | 0.202 | 0.000 | 1.145 | 1.507 | 0.832 |
| Potassium, % | 0.478 | 0.828 | 0.523 | 0.177 | 48.717 | 29.967 | 17.717 | 0.037 | 0.010 | 0.000 | 0.158 | 0.237 | 2.415 | 1.592 | 2.335 |
| Sodium, % | 0.233 | 0.470 | 0.298 | 0.033 | 0.458 | 0.350 | 0.942 | 0.035 | 37.083 | 0.372 | 0.000 | 0.072 | 0.023 | 0.010 | 0.020 |
| Magnesium, % | 0.096 | 0.068 | 0.120 | 0.032 | 0.075 | 0.158 | 0.668 | 0.000 | 0.000 | 0.057 | 0.056 | 0.143 | 0.115 | 0.182 | 0.327 |
| Sulfur, % | 0.39 | 0.73 | 0.44 | 0.06 | 0.12 | 4.21 | 3.97 | 25.02 | 0.00 | 0.13 | 0.12 | 0.40 | 0.44 | 0.48 | 0.41 |
| Iron, mg/kg | 51 | 544 | 60 | 21 | 0 | 36733 | 650 | 0 | 22 | 138 | 14 | 513 | 51 | 63 | 120 |
| Copper, mg/kg | 0.00 | 97.78 | 0.00 | 2.15 | 0.00 | 3553.33 | 0.00 | 0.00 | 0.00 | 0.00 | 3.52 | 8.42 | 5.02 | 22.88 | 17.63 |
| Manganese, mg/kg | 0.00 | 7.85 | 0.00 | 2.20 | 0.00 | 3661.67 | 34.00 | 0.00 | 0.00 | 0.00 | 19.40 | 188.50 | 11.27 | 7.47 | 42.05 |
| Zinc, mg/kg | 56 | 201 | 73 | 7 | 0 | 31250 | 0 | 0 | 12 | 0 | 21 | 84 | 122 | 144 | 54 |
| ^1^ MDC = mechanically deboned low ash chicken; PL = pork liver; GC = ground chicken; GG = guar gum; PC = potassium chloride; MP = mineral premix; KC = kappa carrageenan; TA = taurine; ST = salt; VE = vitamin E 50%, BR = ground brewer’s rice; VP = vitamin premix; LBV = Lalmin B Complex Vitamins; BY = spray dried brewer’s yeast #1064B; EA = BGY Advantage.  ^2^ NFE = nitrogen free extract, calculated (Dry matter basis contents of crude protein, crude fat, crude fiber, and ash subtracted from 100). | | | | | | | | | | | | | | | |
