# Supplementary material for: Composition and thermal processing evaluation of yeast ingredients as thiamin sources compared to a standard vitamin premix for canned cat food
Source: PLoS One. 2022 Aug 2;17(8):e0271600. doi: 10.1371/journal.pone.0271600 (PMC9345363; doi:10.1371/journal.pone.0271600)
Supplement: S2 Table — (DOCX) [file pone.0271600.s002.docx]

| **Table S2: Dry matter basis macronutrient and mineral contents (mean ± standard deviation) of pre-retort and post-retort canned cat foods containing different levels of a vitamin premix and/or a yeast ingredient^1^.** | | | | | | | | |
| --- | --- | --- | --- | --- | --- | --- | --- | --- |
|  | No vitamin premix | | | | Contains vitamin premix | | | |
| Nutrient | NY | LBV | BY | EA | NY | LBV | BY | EA |
|  | ------------------------------------------------------- Pre-retort samples ------------------------------------------------------- | | | | | | | |
| Crude protein, % | 34.6 ± 3.48 | 37.4 ± 4.99 | 45.8 ± 4.36 | 48.9 ± 1.56 | 35.3 ± 3.83 | 34.4 ± 4.95 | 47.0 ± 4.25 | 47.5 ± 1.90 |
| Crude fat, % | 28.1 ± 5.73 | 30.9 ± 6.65 | 26.8 ± 5.45 | 28.3 ± 6.94 | 28.3 ± 4.21 | 24.5 ± 3.40 | 29.3 ± 2.50 | 29.8 ± 4.30 |
| Crude fiber, % | 0.46 ± 0.014 | 0.52 ± 0.023 | 0.85 ± 0.162 | 1.11 ± 0.139 | 0.37 ± 0.337 | 0.58 ± 0.188 | 1.01 ± 0.236 | 1.15 ± 0.093 |
| Ash, % | 6.67 ± 1.918 | 6.56 ± 1.863 | 8.43 ± 2.439 | 7.91 ± 1.691 | 6.14 ± 1.146 | 7.58 ± 2.136 | 7.32 ± 1.171 | 7.97 ± 1.706 |
| NFE^2^, % | 30.3 ± 0.34 | 24.6 ± 7.75 | 18.1 ± 0.74 | 13.8 ± 4.07 | 29.9 ± 5.98 | 32.9 ± 7.89 | 18.1 ± 4.00 | 15.4 ± 3.31 |
| Calcium, % | 1.12 ± 0.442 | 1.00 ± 0.113 | 1.10 ± 0.173 | 1.24 ± 0.285 | 1.06 ± 0.206 | 0.98 ± 0.323 | 1.15 ± 0.258 | 1.26 ± 0.044 |
| Phosphorus, % | 0.895 ± 0.2275 | 0.831 ± 0.0973 | 1.177 ± 0.1080 | 1.045 ± 0.1628 | 0.846 ± 0.1461 | 0.838 ± 0.2043 | 1.150 ± 0.1331 | 1.071 ± 0.0073 |
| Potassium, % | 0.815 ± 0.1824 | 0.770 ± 0.3486 | 1.144 ± 0.1782 | 1.352 ± 0.2929 | 0.721 ± 0.2023 | 0.834 ± 0.2207 | 1.030 ± 0.1901 | 1.420 ± 0.2257 |
| Sodium, % | 0.323 0.0421 | 0.319 ± 0.1067 | 0.324 ± 0.0383 | 0.317 ± 0.0677 | 0.329 ± 0.0536 | 0.318 ± 0.0595 | 0.334 ± 0.0501 | 0.311 ± 0.0488 |
| Magnesium, % | 0.071 ± 0.0110 | 0.065 ± 0.0108 | 0.104 ± 0.0084 | 0.141 ± 0.0154 | 0.066 ± 0.0100 | 0.068 ± 0.0121 | 0.098 ± 0.0060 | 0.146 ± 0.0040 |
| Sulfur, % | 0.41 ± 0.051 | 0.42 ± 0.071 | 0.51 ± 0.060 | 0.52 ± 0.066 | 0.42 ± 0.069 | 0.42 ± 0.075 | 0.51 ± 0.040 | 0.50 ± 0.055 |
| Iron, mg/kg | 278 ± 77.1 | 268 ± 101.5 | 255 ± 81.9 | 295 ± 92.1 | 274 ± 91.1 | 269 ± 91.6 | 277 ± 88.2 | 294 ± 86.4 |
| Copper, mg/kg | 32.38 ± 11.120 | 29.23 ± 16.042 | 36.83 ± 11.188 | 35.37 ± 12.648 | 31.28 ± 11.274 | 31.45 ± 14.080 | 35.62 ± 10.690 | 36.28 ± 11.303 |
| Manganese, mg/kg | 20.07 ± 6.297 | 19.17 ± 6.315 | 14.03 ± 6.388 | 26.63 ± 7.060 | 19.82 ± 6.824 | 20.48 ± 7.476 | 18.65 ± 7.447 | 27.17 ± 6.385 |
| Zinc, mg/kg | 155 ± 51.8 | 161 ± 57.5 | 189 ± 55.0 | 171 ± 56.5 | 168 ± 59.8 | 158 ± 63.6 | 196 ± 59.2 | 171 ± 57.0 |
|  | ------------------------------------------------------ Post-retort samples ------------------------------------------------------ | | | | | | | |
| Crude protein, % | 38.7 ± 1.63 | 40.8 ± 2.06 | 50.7 ± 0.38 | 49.9 ± 2.62 | 43.5 ± 7.73 | 40.2 ± 2.21 | 47.3 ± 5.76 | 49.6 ± 2.28 |
| Crude fat, % | 28.6 ± 8.48 | 28.3 ± 8.63 | 28.6 ± 4.01 | 30.0 ± 6.22 | 31.9 ± 12.50 | 27.6 ± 8.12 | 27.0 ± 1.83 | 30.3 ± 3.85 |
| Crude fiber, % | 0.46 ± 0.285 | 0.72 ± 0.514 | 0.86 ± 0.287 | 1.56 ± 0.267 | 0.85 ± 0.605 | 0.94 ± 0.268 | 0.79 ± 0.222 | 1.93 ± 0.225 |
| Ash, % | 6.14 ± 1.184 | 6.43 ± 0.720 | 7.12 ± 0.909 | 8.31 ± 1.715 | 6.51 ± 1.421 | 6.21 ± 1.065 | 6.84 ± 1.010 | 7.98 ± 1.449 |
| NFE^2^, % | 26.1 ± 5.74 | 23.7 ± 5.38 | 12.7 ± 2.61 | 10.3 ± 1.71 | 17.2 ± 8.13 | 25.1 ± 5.16 | 18.1 ± 5.93 | 10.0 ± 0.83 |
| Calcium, % | 1.22 ± 0.199 | 1.14 ± 0.072 | 0.95 ± 0.433 | 1.30 ± 0.233 | 1.26 ± 0.489 | 1.19 ± 0.217 | 1.12 ± 0.366 | 1.42 ± 0.225 |
| Phosphorus, % | 0.975 ± 0.1348 | 0.958 ± 0.0660 | 1.138 ± 0.2293 | 1.092 ± 0.1393 | 1.080 ± 0.2244 | 0.955 ± 0.1131 | 1.132 ± 0.1821 | 1.134 ± 0.1146 |
| Potassium, % | 0.810 ± 0.2040 | 0.875 ± 0.2017 | 1.186 ± 0.1969 | 1.377 ± 0.2237 | 0.956 ± 0.3560 | 0.856 ± 0.2072 | 1.101 ± 0.1816 | 1.338 ± 0.1146 |
| Sodium, % | 0.323 ± 0.0498 | 0.319 ± 0.0491 | 0.324 ± 0.0502 | 0.317 ± 0.0529 | 0.329 ± 0.0529 | 0.318 ± 0.0566 | 0.334 ± 0.0488 | 0.311 ± 0.0430 |
| Magnesium, % | 0.076 ± 0.0073 | 0.076 ± 0.0048 | 0.104 ± 0.0121 | 0.150 ± 0.0117 | 0.092 ± 0.0221 | 0.077 ± 0.0064 | 0.098 ± 0.0158 | 0.150 ± 0.0090 |
| Sulfur, % | 0.47 ± 0.061 | 0.46 ± 0.051 | 0.54 ± 0.066 | 0.53 ± 0.046 | 0.48 ± 0.093 | 0.46 ± 0.069 | 0.53 ± 0.058 | 0.52 ± 0.038 |
| Iron, mg/kg | 278 ±  90.0 | 268 ±  83.1 | 255 ± 104.0 | 295 ±  95.2 | 274 ± 100.5 | 269 ±  94.6 | 277 ± 103.6 | 294 ±  88.7 |
| Copper, mg/kg | 33.65 ± 11.707 | 33.37 ± 11.098 | 38.92 ± 10.864 | 37.50 ± 10.596 | 36.38 ± 10.570 | 33.42 ± 11.288 | 38.63 ± 13.543 | 35.85 ± 10.078 |
| Manganese, mg/kg | 20.07 ± 7.624 | 19.17 ± 7.255 | 14.03 ± 9.938 | 26.63 ± 8.240 | 19.82 ± 7.907 | 20.48 ± 7.955 | 18.65 ± 9.353 | 27.17 ± 7.558 |
| Zinc, mg/kg | 176 ±  61.3 | 176 ±  55.9 | 179 ±  79.3 | 180 ±  61.4 | 181 ±  69.0 | 176 ±  59.6 | 188 ±  72.6 | 181 ±  58.1 |
| ^1^ NY = no yeast; LBV = Lalmin B-Complex Vitamins; BY = spray-dried brewer’s yeast #1064B; EA = BGYADVANTAGE.  ^2^ NFE = nitrogen free extract, calculated (Dry matter basis contents of crude protein, crude fat, crude fiber, and ash subtracted from 100). | | | | | | | | |
